# Fabrication of multi-parametric platforms based on nanocone arrays for determination of cellular response

**DOI:** 10.3762/bjnano.2.58

**Published:** 2011-09-06

**Authors:** Lindarti Purwaningsih, Tobias Schoen, Tobias Wolfram, Claudia Pacholski, Joachim P Spatz

**Affiliations:** 1 Department of New Materials and Biosystems, Max Planck Institute for Intelligent Systems, Heisenbergstraße 3, 70569 Stuttgart, Germany

**Keywords:** block copolymer nanolithography, cell adhesion, nanostructures, surface chemistry, surface topography

## Abstract

Cellular response to both surface topography and surface chemistry has been studied for several years. However, most of the studies focus on only one of the two parameters and do not consider their possible synergistic effects. Here, we report on a fabrication method for nanostructured surfaces composed of highly ordered arrays of silica nanocones with gold tips. By using a combination of block copolymer nanolithography, electroless deposition, and reactive ion etching several parameters such as structure height and structure distance could easily be adjusted to the desired values. The gold tips allow for easy functionalization of the substrates through a thiol linker system. Improved neural cell adhesion can be obtained and is dependent on the nature of the nanocone surface, thus illustrating the influence of different surface topographies on the nanometer length scale, on a complex cellular behavior such as cell adhesion. Substrate and surface functionality are shown to last over several days, leading to the conclusion that the features of our substrates can also be used for longer term experiments. Finally, initial neural cell adhesion is found to be more prominent on substrates with short intercone distances, which is an important finding for research dealing with the reactions of neuron-like tissue in the immediate moments after direct contact with an implanted surface.

## Introduction

Nanostructured materials for medical applications are intended to be in contact with human tissue and therefore to influence cell function by their surface topography as well as by their surface chemistry. Countless studies on cellular response to nanoscale topographies [1–4], chemical gradients [5–6], and combinations of both [7] have been conducted, which has led to the accumulation of basic knowledge concerning cell morphology changes. However, long-term cellular response to nanostructures has not been understood due to the lack of material integrity. In addition, mainly standard microfabrication techniques including photolithography, wet etching, or reactive ion etching, as well as simple chemical approaches, have been employed for the fabrication of nanostructured materials neglecting the complexity of the biological aspects.

After tremendous work on cellular response to surface features in the micrometer range, such as grooves, ridges and wells, the research focus has shifted to the investigation of the potential of nanostructured materials for controlling cell–surface interactions [8]. For several years experimental studies on the influence of nanoscale topography on cell behavior have been largely obstructed by the lack of nanofabrication techniques to generate functional structures. Recent advances in nanofabrication techniques such as nanoimprint lithography (NIL) [9], nanosphere/colloidal lithography [10], dip pen lithography [11], e-beam lithography [12] have enabled and motivated biomaterial development. However, most of these methods have disadvantages such as high fabrication costs, lengthy preparation times, small-sized nanostructured areas (few square microns) or restricted chemical functionality due to the limited access to composite materials. In particular, the number of material surfaces that offer a tuneable parameter range is insufficient. In spite of the huge progress in material science, chemical and topographical surface gradients have mainly been investigated separately [13] neglecting composite materials such as semiconductor/metal structures [14–17]. Only a few attempts to study cell–surface interactions through topographical and chemical gradients have been reported to date [18]. Representative review articles on neuronal cell response to nanostructured surfaces have been published [19–20]. Despite the enormous effort made in this research area, intelligently designed materials are still required in order to control the interaction between cells and materials, and which can find applications in the fields of tissue engineering, implants, cell-based biosensors, and basic cell biology [21].

In this work, a multiparametric platform for the determination of cellular response has been fabricated by a combination of block copolymer micelle lithography (BCML), electroless deposition (ED) and reactive ion etching (RIE). The resulting highly ordered silica nanocone array with gold tips allows for the investigation of several parameters in cell studies at the same time, that is two- (distance) and three-dimensional (topography) aspects, as well as chemical and biological stimuli. Structures with feature sizes in a range of 50–250 nm for the nanoparticle spacing (i.e., the distance between etched nanostructures) and 10–500 nm for the structural height, can be easily achieved on large areas. Gold nanoparticles on top of the nanostructures allow for spatially resolved functionalization with a variety of biomolecules through simple thiol chemistry. A 3,3'-dithiobis(sulfosuccinimidylpropionate) (DTSSP) linker molecule was used to immobilize laminin, an extracellular matrix, multidomain, trimeric glycoprotein, on the differently structured substrates. Laminin is known to support neural cell adhesion, proliferation, and differentiation. The cell adhesion activity of human neuroblastoma cells (SHSY-5Y) was investigated on these substrates and correlated with topographical features of the nanocone arrays.

## Results and Discussion

**Scheme 1:**
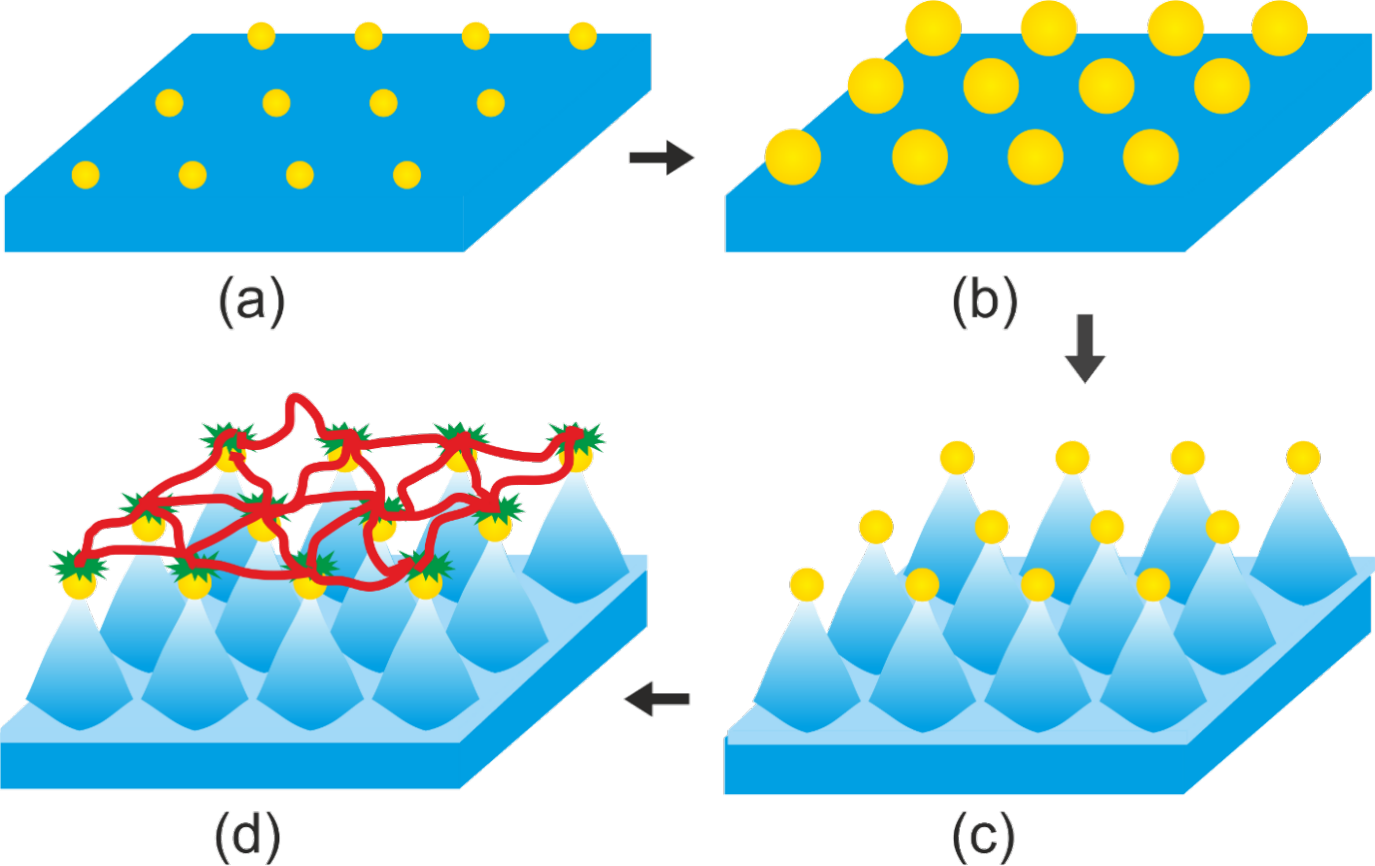
Method used to fabricate silica nanocone arrays with gold functionalized tips. A quasi-hexagonally ordered gold nanoparticle array was deposited on a silica substrate by block copolymer nanolithography (a). Electroless deposition was employed to increase the size of the gold particles (b) before subsequent reactive ion etching was performed (c). The resulting nanostructures can be functionalized with biological active molecules at their gold tips through thiol chemistry. (d) Gold nanocone array whose gold tips have been functionalized with DTSSP and laminin.

Scheme 1 shows the fabrication process for nanocone arrays with gold tips. First, arrays of gold nanoparticles were deposited on a glass surface by diblock copolymer micelle lithography (BCML); this is a versatile technique which allows for the generation of extended quasi-hexagonal arrays of metallic nanoparticles with tuneable interparticle distance (Scheme 1a). Briefly, a diblock copolymer (polystyrene-*block*-poly(2-vinylpyridine), PS-*b*-P2VP) was utilized as a nanoreactor for depositing metallic nanoparticles. A representative SEM image of an as-prepared gold nanoparticle array that should act as a mask upon subsequent reactive ion etching (RIE), is shown in Figure S1a (Supporting Information File 1). However, the small diameters of the gold nanoparticles (1–15 nm) are not sufficient to resist the harsh etching conditions required for the fabrication of the desired topography and chemical functionality (gold on top of the nanocones). To increase the size of the gold nanoparticles, electroless deposition (ED) was used (Scheme 1b) resulting in the generation of nanocone arrays with tuneable topographic features. Electroless deposition is an autocatalytic process that allows for the spatially resolved deposition of metal on to metal surfaces or colloids. Gold nanoparticle arrays with gold particle diameters of approximately 30–45 nm were prepared (Figure S1b). Finally, a reactive ion etching (RIE) process was employed to generate nanocone arrays (Scheme 1c). The etching process was controlled in such a way that gold particles remained on top of the nanocones, in order to provide easily accessible anchor points for biological molecules (Scheme 1d). As expected, the nanostructure height could be controlled by choosing the appropriate etching time. The shape of the etched nanoscale features depends on the etching gas composition as well as on the employed substrate material (glass or fused silica; data not shown).

**Figure 1:**
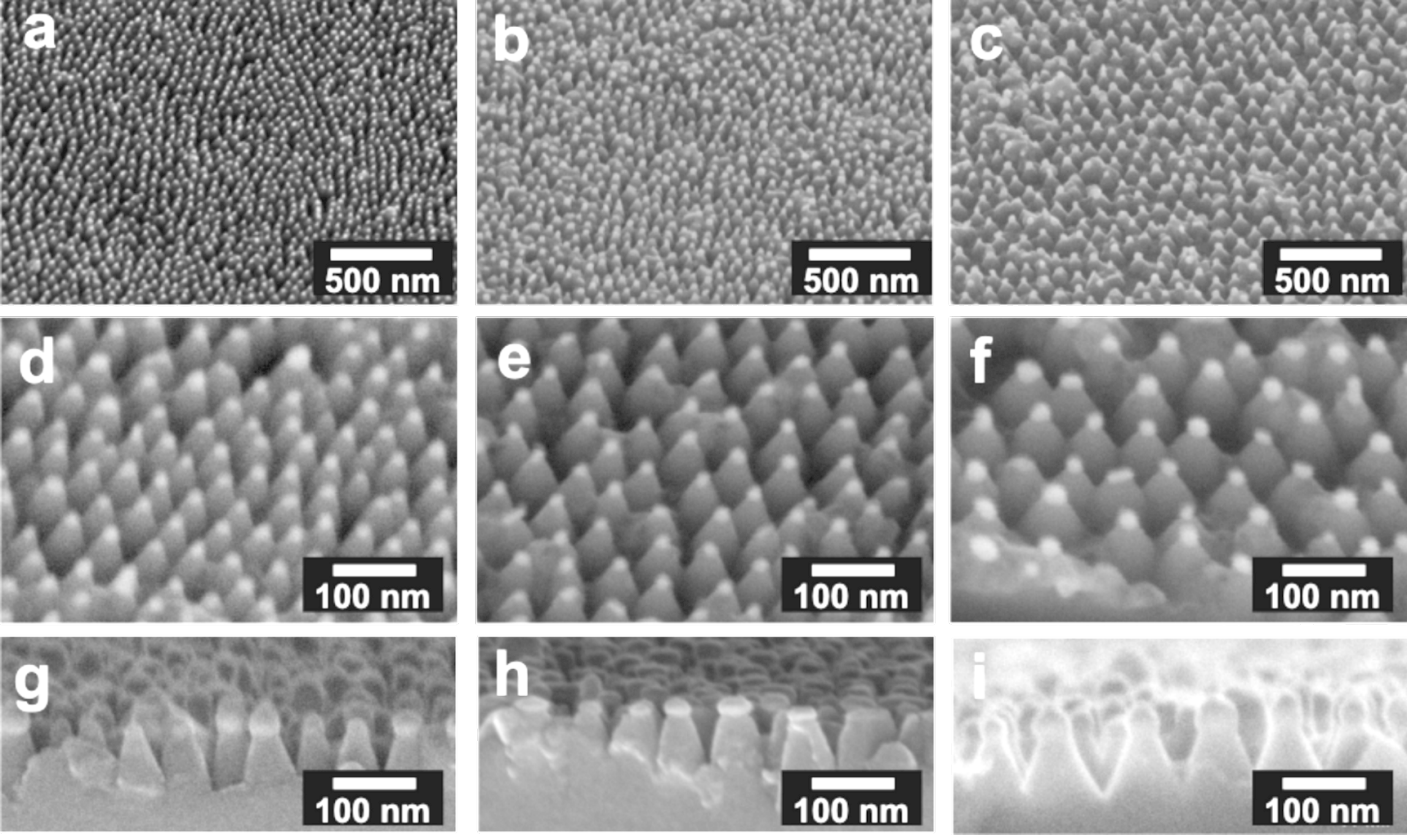
SEM images of the nanocone arrays with gold tips fabricated by a combination of BCML, electroless deposition, and RIE, from three different block copolymer solutions: (PS(501)-*b*-P2VP(323) (first column), PS(1056)-*b*-P2VP(495) (second column), and PS(5355)-*b*-P2VP(714) (third column)). The first row (a–c) shows SEM images of the nanocones taken at a 45° angle from the surface. The second row (d–f) depicts SEM images taken by using an ESB detector showing the compositional intensity differences between the gold particle and amorphous silicon oxide underneath. Finally, the last row (g–i) displays cross-sectional SEM images.

Scanning electron microscopy (SEM) images of the fabricated nanocone arrays with gold tips are displayed in Figure 1 and Figure S1c,d (Supporting Information File 1). Three different diblock copolymers were used for the generation of gold nanoparticle arrays ((PS(501)-*b*-P2VP(323), polymer 501 (first column); PS(1056)-*b*-P2VP(495), polymer 1056 (second column); and PS(5355)-*b*-P2VP(714), polymer 5355 (third column)) in order to tune the interparticle and, consequently, the nanocone spacing, as well as the amount of potential binding sites available in every 1 μm^2^ of the flat surface. Below, each polymer is abbreviated by its PS unit number. Low-magnification side-view SEM images (tilt angle: 45°) of the nanostructures are shown in the first row and confirm the quasi-hexagonal order of the array as well as the variation of the nanocone distance. The location of gold and SiO_2_ in the nanostructures was visualized using an energy selective backscattered (ESB) detector, which gives nanoscale compositional information by contrast differences. Obviously, gold is mainly detected at the tips of the silica nanocones (Figure 1, second row). The height of the different nanostructures is similar and does not depend on the employed diblock copolymer but rather on the etching time (cross-sectional SEM images, third row).

Important parameters of the fabricated nanocone arrays with gold tips are summarized in Table 1. Relevant parameters such as the interparticle distance *d* and the radius of the gold particle *r* were directly extracted from SEM images or deduced by simple calculations (i.e., the number of gold nanoparticles in every 1 μm^2^ surface). In order to obtain reliable data, at least three samples from every batch of the nanostructures were inspected with Inlens, SE2 and ESB detectors. An increase in gold nanoparticle size and interparticle/nanocone distance with increasing diblock copolymer chain length was observed. Consequently, a declining number of gold nanoparticles per μm^2^ of the flat glass surface was obtained with increasing diblock copolymer chain length. The structures had an average areal density of particles of 289 ± 106, 189 ± 32, and 105 ± 20 µm^−2^ for the substrates fabricated from polymer 501, polymer 1056 and polymer 5355, respectively.

**Table 1:** Measured (*r*, *R* and *d*) and calculated (*N*) dimensions of the nanocone arrays fabricated using three block copolymer solutions (PS(501)-*b*-P2VP(323), PS(1056)-*b*-P2VP(495), and PS(5355)-*b*-P2VP(714)).

	PS(501)-*b*-P2VP(323)	PS(1056)-*b*-P2VP(495)	PS(5355)-*b*-P2VP(714)

radius of the gold particle *r* (nm)	13 ± 2	14 ± 3	17 ± 3
radius of the base of the nanocone *R* (nm)	28 ± 5	36 ± 4	42 ± 7
distance between two nanocones *d* (nm)	65 ± 12	78 ± 16	105 ± 26
gold-nanoparticle projected surface density (μm^−2^) after BCML (calculated value)	289 ± 106	189 ± 32	105 ± 20

Contact angle measurements were carried out prior to the surface functionalizations in order to determine the intrinsic hydrophobicity resulting from the nanostructure. Figure S2 (Supporting Information File 1) illustrates the contact angle measurements taken from nanocone arrays prepared from three different diblock copolymer chain lengths, before (first row) and after surface functionalization with DTSSP and laminin (second row). In general, the contact angles and therefore the hydrophobicities of the three fabricated nanostructures are quite similar before functionalization (~60°). Attaching biomolecules to the surfaces lowers the contact angle to values of approximately 20° proving that the hydrophobicity/hydrophilicity of the substrates is a constant parameter in the following cell experiments. The potential of the substrates to support neural cell adhesion was tested with SHSY5Y human neuroblastoma cells.

**Figure 2:**
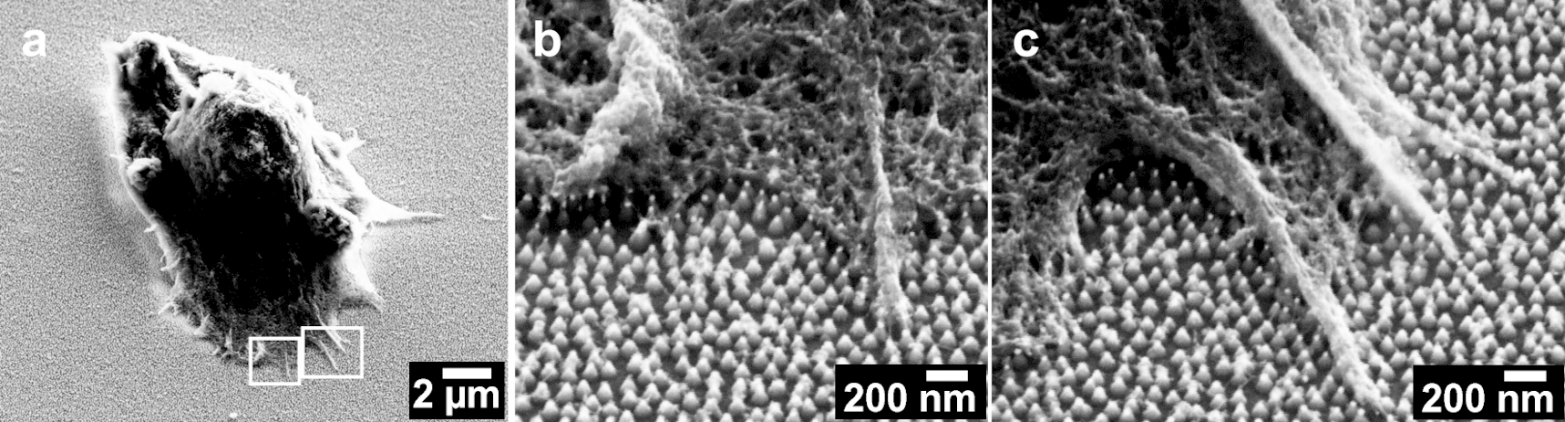
Scanning electron microscopy analysis (45° tilt) of adhering SHSY5Y human neuroblastoma cells. The figure shows a clear adhesion of cellular protrusions to the Au-tipped nanocones. Images were taken at different magnification with a SE2 detector.

Figure 2 shows adhered SHSY5Y human neuroblastoma cells on top of the nanostructured arrays. The gold-tipped nanocones were biofunctionalized with laminin with DTSSP as a thiol-based linker between the gold tips and the protein. Laminin is a protein that mediates cell adhesion and provides a cell survival signal. It was covalently bound to the gold nanoparticles. The line between biofunctionalized nanocone arrays and nonfunctionalized glass is clearly visible in the phase contrast and scanning electron microscopy images (see Figure S3, Supporting Information File 1). The bare glass was completely passivated by silane–PEG2000 and showed little or no cell adhesion. The images shown were taken after 3 hours adhesion time and cells showed protrusion and spreading activity on all substrates.

Figure S4 (Supporting Information File 1) depicts single neural cells and their protrusions, as analyzed by scanning electron microscopy. The substrates were tilted at 45° to make the three dimensional pillar structures visible. For SEM analysis of neural cell adhesion, both Inlens- and SE2-detectors were used. The Inlens detector offers better insights to the cellular details and the SE2-detector gives a better resolution for the nanocone structures. The images show that cellular protrusions adhere on top of the nanocones and not in between. Gold that may have been sputtered back to the surface in small amounts did not lead to effective cell adhesion on the nonfunctionalized side of the substrate. The cell membrane and the cellular protrusions are in close proximity, which is important for the functional aspects of the substrates in possible applications as surfaces for neuro-active implants.

**Figure 3:**
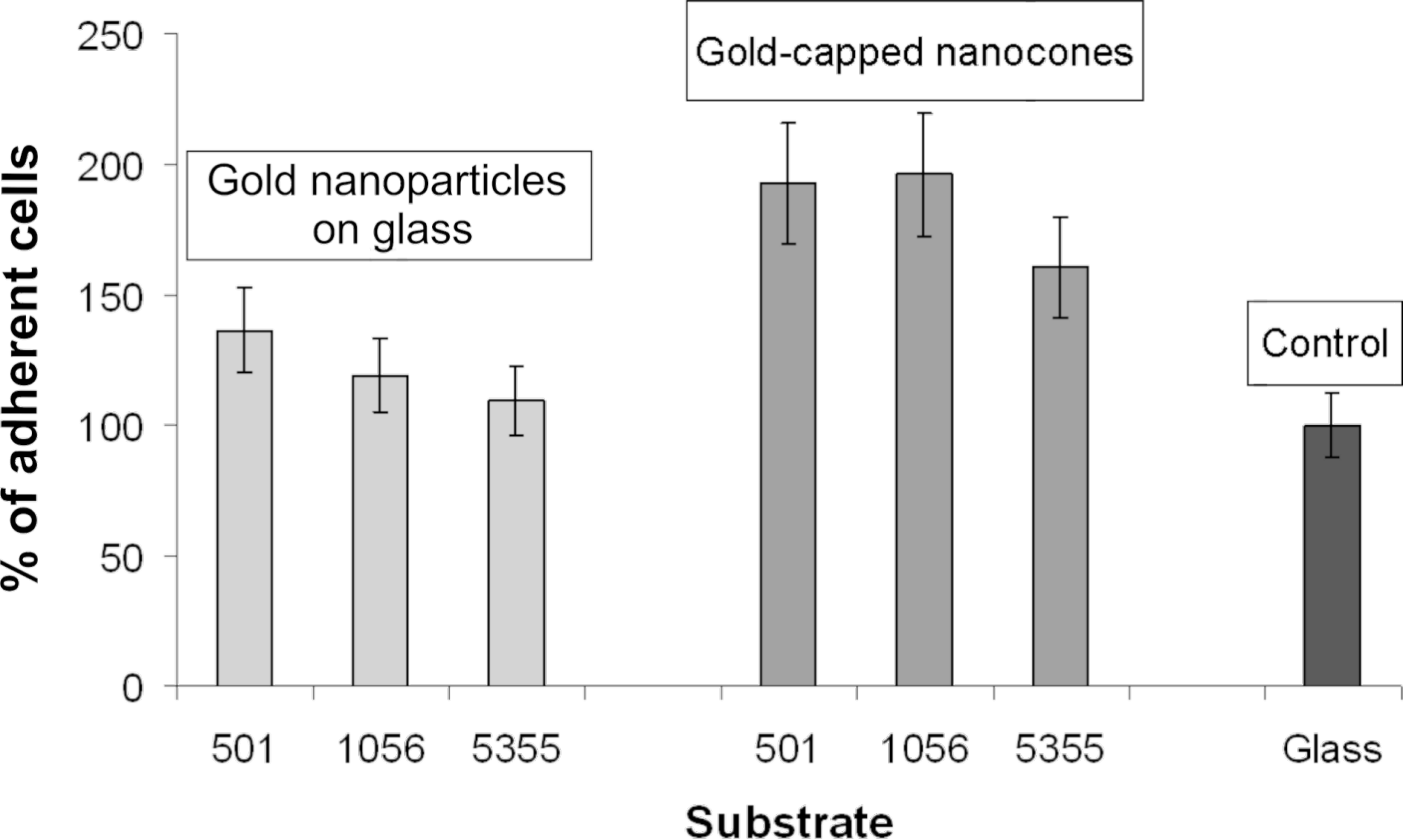
Percentage of adherent cells compared to cell adhesion on control surfaces (glass cover slip coated with laminin, value equals 100%). The nanocone arrays with gold tips show overall higher cell adhesion in comparison to gold nanoparticles on glass or bare glass. Cell adhesion on nanocones is approximately 40% higher compared to flat gold nanoparticles.

Figure 3 shows the quantitative analysis of SHSY5Y-cell adhesion to three different kinds of laminin-functionalized substrate: A flat glass surface, a glass surface decorated with a gold nanoparticle array, and a glass surface structured with an array of gold-capped nanocones. The data shown were extracted from microscopy images taken after 24 hours adhesion time. The gold nanoparticle substrates showed more cell adhesion than did the pure glass surfaces but less cell adhesion than did the nanocone arrays with gold tips. The gold-capped nanocones display overall higher cell adhesion in comparison to flat gold particles or bare glass. Cellular adhesion was ~40% higher on gold-capped nanocones compared to flat gold nanoparticles. Comparison of cell adhesion on substrates fabricated from polymer 501, polymer 1056, and polymer 5355 reveals that cells plated on substrates generated from polymer 501 and polymer 1056 have similar adhesion activities, while cells on the substrates made from the 5355 polymer have slightly less cell adhesion activity in terms of cell numbers. Cellular adhesion is highly influenced by the protein density that is afforded to the cell by the substrate [22–23].

An increased ligand-to-ligand spacing results in an increased distance between the transmembrane proteins of the adherent cells, which in turn results in decreased cellular adhesion. Substrates obtained from polymer 501 and polymer 1056 have similar spacing compared to substrates made from polymer 5355 (Table 1) and thus exhibit similar cell adhesion activities. Our experiments showed that nanocone arrays with gold tips are a suitable tool for higher neural cell adhesion activity and therefore might be interesting as a surface structuring scheme for neuroimplants.

## Experimental

**Synthesis of gold nanoparticle arrays**: The gold nanoparticle arrays are generated on SiO_2_ surfaces by means of the BCML technique as described before [24–26]. Briefly, three different polystyrene(*x*)-*block*-poly(2-vinylpyridine)(*y*) block copolymers were employed: (PS(*x*)-*b*-P2VP(*y*)) with *x* and *y* being 501 and 323; 1056 and 495; and 5355 and 714 respectively, where *x* and *y* represent the number of theoretical repeat units of polystyrene and poly-vinylpyridine, respectively, as calculated by the initial monomer/initiator feed ratio. Except for the polymer solution of *x*, *y* = 501, 323 which was made to a concentration of 5 mg/mL and a loading of 0.4 (defined as *L* = *n*(metal precursor)/*n*(PS-*b*-P2VP)), the other polymer solutions were made to a concentration of 3 mg/mL and a loading of 0.4. The substrates were treated with hydrogen plasma in a PVA TePla plasma system (150 W, 0.4 mbar, and 45 minutes) in order to reduce the metal salts into the corresponding metals and as well as to remove the polymer matrix.

**Gold nanoparticle enlargement by electroless deposition**: The next step is to increase the size of the gold nanoparticles by a published method, known as light-assisted electroless deposition [27–29].

**Reactive Ion Etching**: Afterwards, an RIE process was applied to etch the SiO_2_ layer. A Plasma Lab 80 Plus ICP-RIE system was used, with a mixture of CHF_3_ and CF_4_ gases (at a ratio of 10:1, respectively) at a total pressure of 10 mTorr, a temperature of 20 °C and an RF power of 30 W. The gold particles act as a “mask” for the etch resulting in conical structures with gold particles located at the top. The nanocone arrays with gold tips can later be coated with different molecules such as proteins or antibodies.

**Cell culture and cell adhesion experiments**: Cell culture maintenance for SHSY-5Y neuroblastoma cells was performed as described previously [24]. The substrates for cell adhesion experiments were passivated with silane–PEG2000 as described before [30] and then biofunctionalized with 10 µg/mL laminin-1 with a 25 mM solution of DTSSP (Pierce, USA) in PBS as a linker between gold and laminin. Laminin-coated glass cover slips (control) were fabricated by a standard protocol [31]. SHSY5Y human neuroblastoma cells were then incubated on the substrates for 3–24 h at 37 °C and 5% CO_2_ at a cell density of 80,000 cells/mL. The cells were fixed with 4% para-formaldehyde and analyzed by phase contrast microscopy (Zeiss Axiovert 40 CFL microscope). The average cell counts per measured field with 10× magnification objective normalized to glass for each substrate, offer the possibility to directly compare each substrate and deduct its adhesion activity. After critical point drying (Baltec CPD 030 critical point dryer), the substrates were coated with carbon by means of a Med 020 Coating System (Leica Microsystems) and finally examined by scanning electron microscopy (Zeiss Gemini Ultra-55).

## Supporting Information

Supporting Information features additional images and illustrations of the nano-arrays and the adhered cells.

File 1 Additional images and illustrations.
