# Supplementary material for: Fabrication of multi-parametric platforms based on nanocone arrays for determination of cellular response
Source: Beilstein J Nanotechnol. 2011 Sep 6;2:545–51. doi: 10.3762/bjnano.2.58 (PMC3190624; doi:10.3762/bjnano.2.58)
Supplement: File 1 — Additional images and illustrations. [file Beilstein_J_Nanotechnol-02-545-s001.pdf]

Supporting Information

for

Fabrication of multi-parametric platforms based on nanocone arrays for determination of cellular response

Lindarti Purwaningsih‡, Tobias Schoen‡, Tobias Wolfram, Claudia Pacholski and Joachim P. Spatz*

Address: Department of New Materials and Biosystems, Max Planck Institute for Intelligent Systems, Heisenbergstraße 3, 70569 Stuttgart, Germany

Email: Joachim P. Spatz* - spatz@mf.mpg.de

* Corresponding author

‡ Equal contributor

Additional images and illustrations

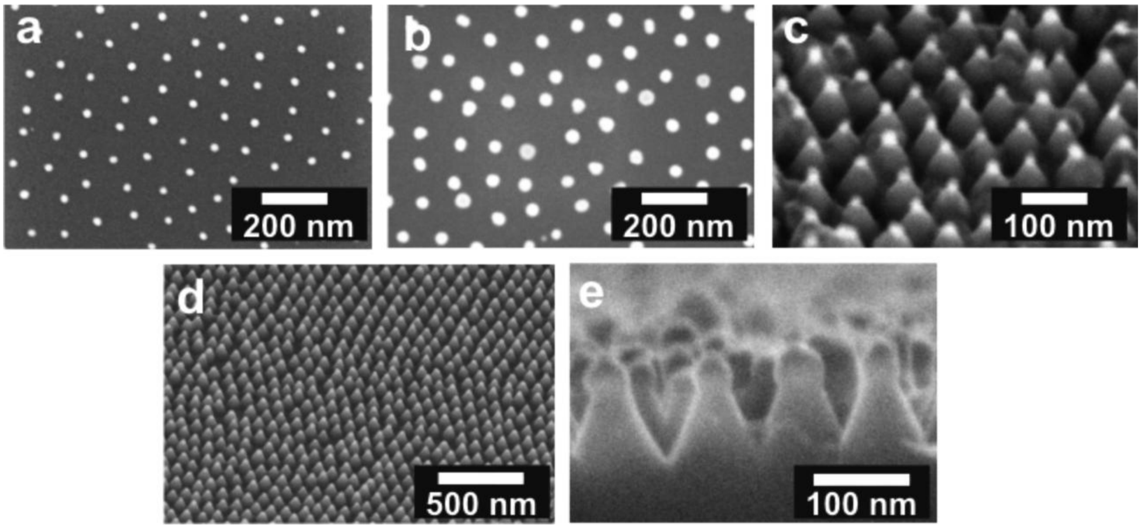

Figure S1: SEM images of a quasi-hexagonally ordered gold nanoparticle array before electroless deposition (a), after electroless deposition (b), and cone-shaped structure after reactive ion etching (c). An array of conical structures with gold particles located on top (d) and a cross sectional SEM image of the nanostructure (e).

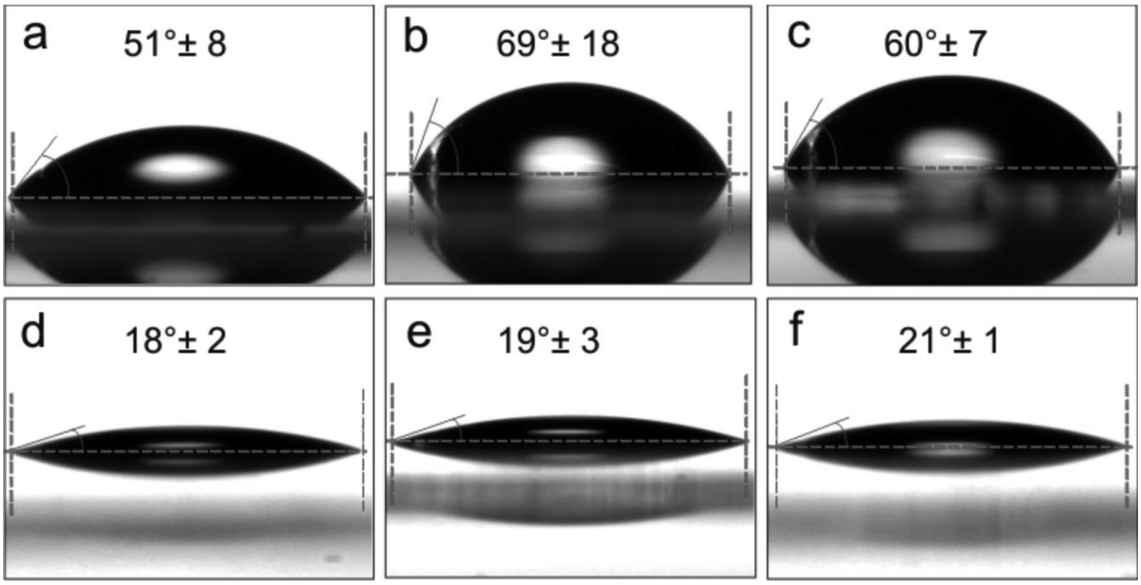

Figure S2: Contact angle measurements of nanostructured surfaces prepared using three different block copolymers before (first row) and after the surface functionalizations with DTSSP and laminin (second row). The contact angles of the substrates fabricated from polymer 501 (a), polymer 1056 (b) and polymer 5355 (c) before functionalization were 51 ± 8° (a), 69 ± 18° (b) and 60 ± 7° (c). After functionalization with DTSSP and laminin the values changed to 18 ± 2°, 19 ± 3° and 21 ± 1° (d–f), respectively.

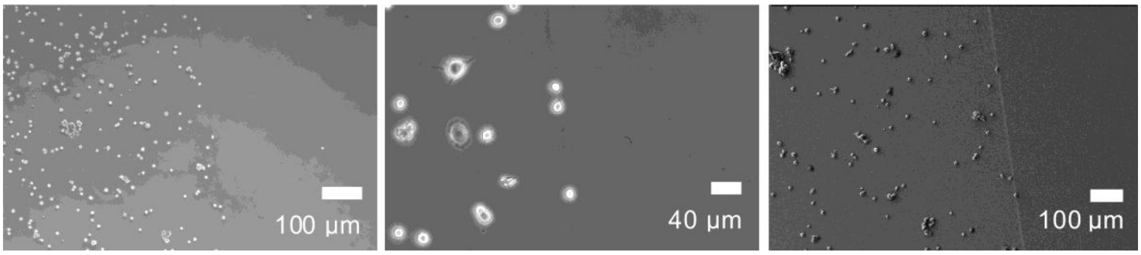

Figure S3: SHSY5Y human neuroblastoma cells on top of nanocones that were biofunctionalized with Laminin after 3 hours incubation time. The unfunctionalized area between the glass was passivated with silane–PEG2000. The interface between biofunctionalized (left) and unfunctionalized (right) areas can easily been distinguished by the absence of cells on the unfunctionalized area.

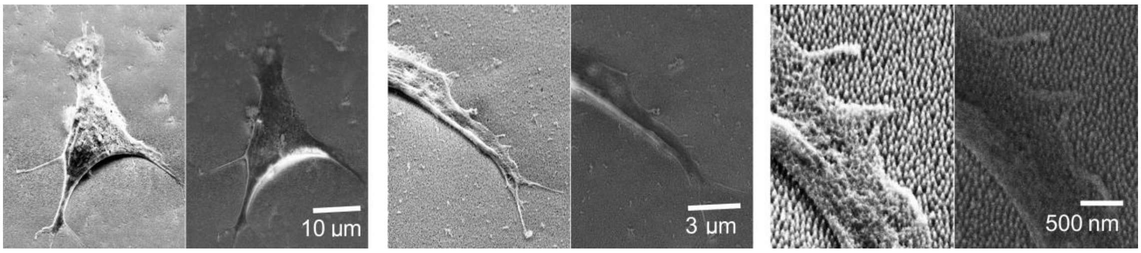

Figure S4: SEM images of SHSY5Y human neuroblastoma cells on functionalized nanocone arrays taken with both SE2- and inlens-detector. Cellular details are better resolved with the inlens-detector, whereas the SE2-detector offers a better resolution of the nanocone.
